# The practice and determinants of ambulance service utilization in pre-hospital settings, Jimma City, Ethiopia

**DOI:** 10.1186/s12873-024-00999-8

**Published:** 2024-05-12

**Authors:** Mohammed Ahmed Adem, Zewdu Baye Tezera, Chilot Desta Agegnehu

**Affiliations:** 1 Department of Emergency and Critical Care, School of Nursing, Faculty of Health Sciences, Institute of Health, Jimma University main campus, 378 Jimma, Ethiopia; 2 School of Nursing, College of Medicine and Health Sciences, University of Gondar, 196, Gondar, Ethiopia

**Keywords:** Ambulance, Pre-hospital care, Emergency medical service (EMS), Ethiopia

## Abstract

**Background:**

In pre-hospital setting, ambulance provides emergency care and means of transport to arrive at appropriate health centers are as vital as in-hospital care, especially, in developing countries. Accordingly, Ethiopia has made several efforts to improve accessibility of ambulances services in prehospital care system that improves the quality of basic emergency care. Yet, being a recent phenomenon in Ethiopia, empirical studies are inadequate with regard to the practice and determinants of ambulance service utilization in pre-hospital settings. Hence, this study aimed to assess the ambulance service utilization and its determinants among patients admitted to the Emergency Departments (EDs) within the context of pre-hospital care system in public hospitals of Jimma City.

**Method:**

A cross-sectional study design was used to capture quantitative data in the study area from June to July 2022. A systematic sampling technique was used to select 451 participants. Interviewer-administered questionnaire was used to collect data. Data analysis was done using SPSS version 26.0; descriptive and logistic regressions were done, where statistical significance was determined at *p* < 0.05.

**Results:**

Ambulance service was rendered to bring about 39.5% (of total sample, 451) patients to hospitals. The distribution of service by severity of illnesses was 48.7% among high, and 39.4% among moderately acute cases. The major determinants of ambulance service utilization were: *service time* (with AOR, 0.35, 95%CI, 0.2-0.6 for those admitted to ED in the morning, and AOR, 2.36, 95%CI, 1.3-4.4 for those at night); *referral source* (with AOR, 0.2, 95%CI, 0.1-0.4 among the self-referrals); *mental status* (with AOR, 1.9, 95%CI, 1-3.5 where change in the level of consciousness is observed); *first responder* (AOR, 6.3 95%CI, 1.5-26 where first responders were the police, and AOR, 3.4, 95%C1, 1.7-6.6 in case of bystanders); *distance to hospital* (with AOR,0.37, 95%CI, 0.2-0.7 among the patients within ≤15km radius); and *prior experience* in ambulance use (with AOR, 4.1,95%CI, 2.4-7).

**Conclusion:**

Although the utilization of ambulance in pre-hospital settings was, generally, good in Jimma City; lower levels of service use among patients in more acute health conditions is problematic. Community-based emergency care should be enhanced to improve the knowledge and use of ambulance services.

## Background

Pre-hospital care is out-of-hospital emergency care provided by trained healthcare with the ability to provide transportation to healthcare facility [1, 2]. It includes medical treatments in pre-hospital settings and means of emergency patient transportation to appropriate healthcare centers[3]. Therefore, pre-hospital care is regarded as vital as in-hospital medication, because its contribution is significant including survival benefits for people who are critically ill and prevention of complications in time-sensitive situations [4, 5].

Historically, the health care systems in many developing countries have focused on treating communicable diseases [3]. However, the steady rise in the burden of emergency conditions due to high Road Traffic Crash (RTC) and life-threatening non-communicable illnesses have led to the acknowledgment of the importance of pre-hospital care intervention to mitigate the fatalities of the problems [6, 7]. According to the World Bank report, most of the mortality cases, including about 54% of deaths and 91% years of life lost, which occur in the pre-hospital settings, could have been prevented in the presence of robust emergency care in Low- and Middle-Income Countries (LMICs) [3].

Cognizant of this fact, pre-hospital care systems are rapidly developing in LMIC, especially in remote areas to reduce morbidity and mortality in the region [8, 9]. Yet, empirical evidence shows that the level of ambulance utilization to take patients to emergency departments (ED) of nearby health center is very low across LMICs, ranging from 4% to 37%, which implies that majority of the patients suffer from shortage of ambulance services [10–17].

In the context of Africa, the use of ambulance service in pre-hospital settings is even far worse, because of multifaced challenges and barriers. A study conducted in Ghana revealed that only 5% of patients use ambulance to arrive EDs although about 75% of people experience medical and traumatic emergencies [18]. Scholarly works indicate that, in Africa, the reason for low pre-hospital care were doe to uncoordinated pre-hospital care, lack of trained human and funding resources, poor road infrastructures, location and response time of ambulance services, low level of community engagement and misunderstanding of the services [18–23].

In the same vein, Ethiopia has recently made several efforts to implement world health organization of basic emergency care by improving and developing a pre-hospital care system as a mechanism to deal with the stress of emergencies arising from high RTC, communicable and non-communicable health deficiencies, disproportionately affecting the productive age groups, resulting in early death and disability [15, 24, 25]. The country, is currently pursuing the “two-tiered” pre-hospital emergency care systems, that primarily aim at rapidly bringing patients to hospital to offer basic emergency medical services by trained personnel [26]. In this regard, the Ministry of Health has been working to improve the number of ambulances, manpower, community participation, and raise public awareness of access to and use of ambulance services [27]. Nevertheless, pre-hospital care in Ethiopia has, previously, been limited to the capital city, and currently, the services are rapidly becoming an important part of universal health care, and extending to the areas with a high burden of emergency diseases, RTC and most populous cities [24, 26].

Jimma City is one of the oldest, rapidly urbanizing and most populated urban areas heavily burdened with emergency conditions, where emergency health care is provided at two public hospitals serving the community as referral health care centers: namely, Jimma University Medical Center (JUMC) and Shenen Gibe Hospital (SGH). As of October 2022, JUMC, in collaboration with Jimma Health Care Office and the Ministry of Health, has established a hospital-based Emergency Medical Services (EMS) known as “*Aayyoo* *Ambulance*”. Currently, the service has only one dispatch center that operates on the four digits ‘6238’ toll-free call number. Since its establishment, the center has been providing 24-hour/7-days free charge of services to the population of Jimma City and its vicinities.

While quantitative evidence is inadequate with regard to the pre-hospital care in Ethiopia, Jimma City included, it is reported that patients with emergency illnesses often arrive too late to EDs, sometimes after developing complications, despite improvements in the quality and availability of ambulance services. This rationalizes studying ambulance service utilization and associated factors as an important element of the broader health care system that determines the pre-hospital emergency outcomes. Therefore, this study is designed with the aim of assessing the status of ambulance service use in the pre-hospital setting among patients visited at adult EDs, and to identify the determinant factors associated with service utilization in the study area.

## Methods

### Study design, and setting

This study has used a quantitative research approach, which is common in medical and public health researches. More specifically, the research employed a cross-sectional study design, a research mechanism that involves the collection of data from a population at a single point in time that offers a valuable insight into population characteristics in examining individuals or groups relationships or differences [28]. Accordingly, cross-sectional study design was used to capture relevant quantitative data from various strata of population at a particular period in the study area in such a way to examine ambulance service utilization and its determinants. In line with the objective of the research, the data was collected, during June – July 2022, from patients admitted to the Emergency Departments (EDs) in JUMC and SGH, the only public hospitals rendering emergency medical services in Jimma City, southwest Oromia, Ethiopia. The two selected public hospitals were located in different part of the city, providing emergency services and trauma care. Accordingly, the data was collected, from June to July 2022, in the two referral hospitals of the city.

### Study population

The study populations were all patients visited at the adult EDs with time-sensitive conditions and all trauma cases that passed through a single point of triage zone. Patients with emergency cases of inter-facility transfer from another health center and trauma cases of pediatrics were also included. Those patients who were unable to provide responses due to their own health conditions or the absence of attendants and normal laboring mothers were excluded.

### Sample size and sampling technique

The sample size was determined by using a single population proportion statistical formula by taking *p*=0.203, where *p* is the proportion of emergency cases in which ambulance was utilized [14], with 95% of confidence interval (CI) and 3% margin of error. On the basis of previous studies, the average monthly patients admitted to the EDs of the two hospitals were found to be 1,105, which was less than 10,000. Hence, the correction formula was used by adding 10% of non-response rate that made the sample size 468.

The study participants were proportionally drawn from each hospital, 65% from JUMC and 35% from SGH, by using the average patient flow with emergency cases according to the monthly performance indicators sought from the Emergency Head Department of the hospitals. Then, a systematic random sampling technique was used to select the desired study participants from the two hospitals by using a sequential number of medical records as a sampling frame.

### Data collection tools and procedures

Questionnaire was employed to obtain the required primary data from study participants based on standard survey tools of previous studies [18]. The questionnaire was translated to the most widely used local languages (*Amharic* and *Afan Oromo*), later, translated back to English for the purpose of coherence and consistency. The questionnaire consisted of three sections: the socio-demographic characteristics, the clinical characteristics, and the types of pre-hospital ambulance services utilized including the reasons as well as treatments. In the study setup, the majority of health workers are structured into three work shift patterns, consisting of a 6-hour morning shift from 8:00 a.m. to 2:00 p.m., 6-hour afternoon shift from 2:00 p.m. to 8:00 p.m., and a night shift from 8:00 p.m. to 8:00 a.m. Accordingly, the time of patient arrivals to the EDs was analyzed to determine ambulance utilization [29]. The average distance of patient traveled from the scene to the location of the EMS was measured according to the oral responses of the participants[30]. The outcome variable was, then, categorized as an ambulance and non-ambulance based on the patient’s mode of arrival to the EDs.

Before the actual data was collected, a pre-test was done by using 5% of sample size on target patients and/or individuals who accompanied them to the EDs of Tibebe Ghion referral hospitals of Bahir Dar City, which have similar EMS, to improve wording, design and structure of the questionnaire. The validity of the tools was examined by the research experts. Finally, the actual data was collected by enumerators who recorded the oral responses from patients or their attendants to the questionnaire. The enumerators were among the experienced health care workers specifically hired and trained for the study purpose. In cases where the patients were respondents, the responses were collected only after ensuring that the patients were clinically stable as the acceptability of such procedure had acceptable responses [21].

### Data quality management and analysis

Quality of the data was ensured manually by checking the completeness and consistency before data entry into *Epi-data 4.6* software. The data was, then, exported to *SPSS version 26* software for further cleaning and analysis. Descriptive statistics were done for all variables. Factors associated with patients’ use or non-use of ambulance service were compared based on Pearson’s *Chi-square* test at 0.05 level of significance. The assumption of model goodness of fit was tested by using the Hosmer and Lameshow test at chi-square test 6.06 and p-value 0.64. All variables with a p-value <0.25 in bivariate regression analysis were included for multivariable analysis to control the possible confounders and look at the factors associated with the outcome of ambulance use. Moreover, Crude Odds Ratio (COR), Adjusted Odds Ratio (AOR) and CI were used to determine the strength of associations between dependent and independent variables.

### Ethical consideration

An official letter of ethical clearance with a reference number ‘S/N 254/2014’ was obtained from the College of Medicine and Health Science institutional review board (IRB) of the University of Gondar. Permission was received from emergency center of the host hospitals to collect the data. Informed consent was obtained from the legal guardians of the participants for those who cannot read and write, as well as the minors. The autonomy and confidentiality of the study participants were preserved and secured during the study and dissemination of the results. In addition, informed consent was cordially obtained directly from the study participants.

## Results

### Socio-demographic characteristics of respondents

During the conduct of the study, 468 participants were approached, out of which 451 provide a complete response to the survey questions, making the overall response rate 96.4%. Of the 451 participants with complete survey responses, 123(27.3%) were patients; whereas, 311(69%) were relatives of the patients and 17(3.7%) were policemen and bystanders who brought the patient to hospitals. The gender distribution of participants revealed that 252(55.9%) were male. Nearly half, 220(48.8%), of the patients were aged between 25-44 years, with a mean average age of 37.6 where the standard deviation ± 16.2. As far as the educational profile of respondents is concerned, it was found that only 64(14.2%) of participants attained college education and above.

More than half, 250(55.4%), of the respondents had no health care insurance, and 418(92.7%) didn’t own a car or motorcycles for transportation Table 1.

**Table 1:** Socio-demographic characteristics of the respondents visited at EDs of public hospitals in Jimma city, Ethiopia, June 2022. (*n*=451)

**No**	**Variables**	**Responses**	**Frequency**	**Percentage**
1.	Sex	Male	252	55.9%
		Female	199	44.1%
2.	Age	13-24	91	20.2%
		25-44	220	48.8%
		45-60	90	20.0%
		>60	50	11.1%
3.	Educational status	Cannot read and write	143	31.7%
		Can read and write	88	19.5%
		Primary education	85	18.8%
		Secondary education	71	15.7%
		College and Above	64	14.2%
4.	Mobile phone ownership	Yes	301	66.7%
		No	150	33.3%
5.	Health care insurance coverage	Yes	201	44.6%
		No	250	55.4%
6.	Own a car/motorcycle	Yes	33	7.3%
		No	418	92.7%
7.	Heard about Ambulance service	Yes	438	97.1%
		No	13	2.9%
8.				
9.				

### Condition of emergency, mode of arrival and clinical characteristics of patients

Looking at the types of emergency cases among patients visited at the EDs, 331(73.4%) were medical emergency cases, whereas the remaining 120(26.6%) consisted of traumatic emergencies.

Data results also revealed that 312(69.2%) patients reported an acute level of illness upon arrival at the EDs, where 183(58.7%) of them relied on non-ambulance modes of transportation including regular and three-wheeled taxies, public transportation, automobiles of government institutions, and others. Even though 115(25.5%) of the patients were triaged as highly acute cases upon their arrival to the EDs of the hospitals, only 56(48.7%) of them arrived by ambulance. Moreover, the majority, 343(76.1%), of emergency cases occurred at home; whereas, schools, recreation and work places shared 35(7.8%) of the cases (Table 2).

**Table 2:** Condition of emergency, mode of arrival and clinical characteristics of patients visited at the EDs of public hospitals in Jimma city, Ethiopia, June 2022. (*n*=451)

**No**	**Variables**	**Responses**	**Ambulance**	**Non-Ambulance**	**Total**
			**No (%)**	**No (%)**	**(*N*=451)**
1.	Type of emergency illnesses	Medical illnesses	117(35.3%)	214(64.7%)	331
		Trauma	61(50.8%)	59(49.2%)	120
2.					
3.					
4.	Illness onset	Acute	129(41.3%)	183(58.7%)	312
		Non-acute	49(35.3%)	90(64.7%)	139
5.					
6.	Triage acuity	Low acuity	61(33.7%)	120(66.3%)	181
		Moderate acuity	61(39.4%)	94(60.6%)	155
		High acuity	56(48.7%)	59(51.3%)	115
7.	Patient’s companion	Family/relatives	163(41.6%)	229(58.4%)	392
		Bystander/Police	15(25.4%)	44(74.6%)	59
8.	Emergency scene	Home	125(36.4%)	218(63.6%)	343
		Street	40(54.8%)	33(45.2%)	73
		Others	13(37.1%)	22(62.9%)	35
9.					

### Ambulance utilization in the pre-hospital care during emergency conditions

This study revealed that 178(39.5%) of the respondents (with 95%CI: 35%, 44%) had utilized ambulances service as part of pre-hospital emergency care to arrive at the EDs. Among those patients who had utilized ambulance, only 35(19.7%) have got primary ambulance service from the scene of emergency to the hospital, whereas 143(80.3%) of the patients arrived *via* inter-facility referral ambulance services. In more than half, 93(52.2%), of the cases, ambulance services were activated by health care professionals. It happened that the reason for ambulance service usage among 105(59.0%) patients was due to the decision of health workers, whereas in other 5(2.4%) cases, it was because of the decision of family or bystander, besides the perceptions involving cost and time factors associated with the treatment associated with the free-service and shorter waiting time that the ambulance services offers.

Out of 178(39.5%) ambulance utilizers, only 63(35.4%) of patients received treatment on the way to the hospitals, in which control of bleeding was found to be the major treatment that accounted for 21 (32.8%) cases (Table 3).

**Table 3:** Types and reasons of ambulance usage along with treatments received before arriving at the EDs of public hospitals in Jimma city, Ethiopia, June 2022. (*n*=178)

**No**	**Types and service provided**		**Frequency**	**Percentage**
1.	Type of ambulance service	Primary ambulance response (i.e. from the scene)	35	19.7%
		Inter-facility ambulance service	143	80.3%
2.	Ambulance service activators	Patient	3	1.7%
		Bystander	33	18.5%
		Family members	43	24.2%
		Police	6	3.4%
		Health professional	93	52.2%
3.	Ambulance service provider	Public health care facility	134	75.3%
		Private facility	4	2.2%
		Public dispatch center	12	6.7%
		Red cross	16	9.0%
		Municipality	12	6.7%
4.	Reason for ambulance utilization	Health workers’ decision	105	59.0%
		Shorter time to hospital	49	27.5%
		To get immediate treatment	38	21.3%
		Severity of the illness	32	18.0%
		Others	5	2.8%
5.	Received care in ambulance	Yes	63	35.4%
		No	115	64.6%
6.	Type of treatments obtained in ambulance (*n*=63)	Bleeding control	21	32.8%
		Immobilization	17	26.6%
		Pain management	15	23.4%
		Fluid/IV care	11	17.2%
		Oxygen care	18	28.1%

### Factors associated with ambulance service utilization

After bi-variable and multivariable logistic regression analyses were done, the following variables were identified, based on their statistical significance. The result of multi-variable logistic regression analyses suggested that the time of arrival at the EDs, source of referral, mental status of the patients, first responder, distance from hospital and previous experience were the major variable significantly associated with the utilization of ambulance in the pre–hospital emergency conditions at *p*-value <0.05 (Table 4).

**Table 4:** Factors associated with ambulance utilization among patients visited at the EDs of public hospitals in Jimma city, Ethiopia, June 2022. (*n*=451)

**No**	**Variables**		**Ambulance**	**Non-Ambulance**	**COR(95%CI)**	**AOR(95%CI)**
1.	Time of arrival at ED	Night	68	42	2.15(1.31-3.53)	2.36(1.26-4.44)**
		Morning	40	138	0.39(0.24-0.62)	0.35(0.19-0.63)**
		Afternoon	70	93	1	1
2.	Referral source	Self-referred	18	118	0.15(0.09-0.26)	0.19(0.10-0.37)**
		Private health facility	9	5	1.79(0.59-5.46)	3.45(0.77-15.41)
		Government health facility	151	150	1	1
3.	Mental state	Fully alert	94	193	1	1
		Confused	61	65	1.93(1.26-2.96)	1.87(1.0-3.47)*
		Comatose	23	15	3.15(1.57-6.31)	2.44(0.93-6.4)
4.	First Responder	Patient	79	169	1	1
		Bystander	67	57	2.5(1.62-3.94)	3.37(1.71-6.64)**
		Police	13	9	3.09(1.27-7.53)	6.32(1.5-26.69)**
		Family	19	38	1.07(0.58-1.98)	0.97(0.45-2.11)
5.	Distance to hospital	≤15km	42	121	0.39(0.26-0.59)	**0.37(0.19-0.68**)**
		≥16km	136	152	1	1
6.	Prior use of ambulance	Yes	87	44	4.98(3.23-7.70)	**4.1(2.38-7.01**)**
		No	91	229	1	1

Statistical significance levels: * *p*<0.05; and, **p<0.01; Crude Odds Ratio (COR) and Adjusted Odds Ratio (AOD)

## Discussion

This study has assessed the status of pre-hospital ambulance service utilization during emergency health conditions among patients visited at the adult EDs of public hospitals in Jimma City, southwest of Ethiopia. Based on the data results, several factors were found to be associated with ambulance service utilization in pre-hospital settings.

As alluded to, the pre-hospital ambulance service utilization was found to be 39.5%, with 95% of CI (35%, 44%). This meant that ambulance service use in pre-hospital emergency conditions was higher in the study area if compared to the findings of previous studies conducted in Mekele city [16] and the capital, Addis Ababa, of Ethiopia [14, 15]. Similarly, previous studies in LMIC including the studies undertaken in Ghana [10], Pakistan [11], Nepal [31], and Lebanon [12] have reported a lower proportion of ambulance service utilization in emergency health situations.

The higher level of ambulance usage in the study area compared to other parts of Ethiopia or other LMICs was attributed to the recent attention given to pre-hospital care services in the Ethiopia that led to the establishment of an EMS system in Jimma city and awareness raising activities undertaken by the Ministry of Health. Moreover, launching of an EMS agency that operates *via* a toll-free call number in Jimma city also facilitated ease of access to ambulance service thereby improving utilization of ambulances.

This study also found that the night time arrivals at the EDs, altered level of consciousness, being referrals from health care facility, first response (extrication) from the scene by bystander/police and previous experience in utilization were positively associated with ambulance service. On the contrary, arriving at the EDs in the morning time, self-referral and patients’ travel distance (≤15km) were negatively associated with ambulance utilization.

The analyses of this study showed that the arrival time of patients to the EDs was one of the main determinant factors for ambulance service utilization. Patients who arrived at night were 2.4 times more likely to use ambulances, but patients who arrived in the morning were 65% less likely to use ambulance [29, 32]. The result of this study also pointed out that, in Ethiopia, specifically in the study area, the mode of transportation was more available during the day time than night so that ambulance services were utilized more when other modes of transportation was inaccessible.

The utilization of the ambulance was higher in cases of inter-facility health care. This was evident as the use of ambulances among the self-referred patients was 80% less likely compared to those patients referred from the government health care facilities. Patients with an altered level of consciousness upon arrival to the EDs were twice more likely to use ambulances than alert patients. These findings were similar to other studies conducted in Africa such as Ghana [10]. High level of ambulance use among the patients of inter-facility health service was due to the fact that the majority (69.8%) of the study participants were referred from government health centers, and ambulance service utilization was mostly the decision of health care workers, besides the severity of the patient’s illness.

The ambulance service utilization during emergency conditions was also influenced by the location of the emergency scene and first aid response given by the bystanders. As evidenced from the results, ambulance service utilization among the patients extricated from the scene by the bystanders were 3.4 times higher, and those by police were 6.3 times more ambulances utilized than those patients extricated by themselves. This finding suggested that better awareness raising activities about ambulance usage have enhanced the contribution of bystanders in the pre-hospital care, especially in improving ambulance service use and the outcome of emergency illnesses.

Prior experience of ambulance use was positively associated with ambulance utilization. This result was consistent with the findings of previous studies that revealed the relevance of prior history with regard to ambulance utilization as in Addis Ababa [14], and Ghana [18]. This indicated that the familiarity with, and satisfaction of patients with the ambulance service was crucial.

The distance from hospitals was also another major factor for ambulance service utilization, as those patients who travelled a distance of more than 16km were 2.7 times more likely to use ambulance. This finding was consistent with the study conducted in Mekele [20], which revealed that patients’ travel distance of more than 30 minutes was more likely to result in the utilization of ambulance services. But, it was contrary to the study conducted in Thailand [31], which indicated that ≤15km distance from the health center was more likely to lead to ambulance service usage. This disparity was, once again, explained by the fact that the majority of participants in this study were inter-facility referred patients, but also because patients from the urban area utilized other non-ambulance modes of transportation.

### Limitation

This study was conducted in Jimma city, where there was an organized EMS system, that resulted in the ease of accessibility of the ambulance service. Hence, the results of this study might not be replicated to other parts of Ethiopia because the EMS is not available in many other parts of the country.

Besides, some patients who came to the EDs by ambulance were excluded because they were not accompanied by any relative (or attendant) to provide the required responses or else the patient him/herself was unable to provide complete information during data collection. Moreover, labouring mothers were excluded, though the expectation of ambulance usage is high, for the obvious reason that they usually get the necessary medical care in the labour room, while this study primarily focused on the patients visited at the adults’ EDs of the two public hospitals in the study area.

## Conclusion

The ambulance service utilization among patients with emergency health conditions visited at adult EDs of public hospitals in Jimma City was low in proportion against the severity of the illnesses. More than half of patients with emergency health condition including acute illnesses rather made use of non-ambulance modes of transportation to the hospitals. This study also identified that arrival at the EDs at night, altered level of mental status, being referred from health care facility, first response given by bystander and previous experience of service utilization were positively associated with service utilization. While the morning time arrivals at the EDs, self-referrals and patients’ travelled (≤15km) distance to hospitals were negatively associated with ambulance utilization. Therefore, community targeted pre-hospital care should be enhanced to improve the knowledge about the advantages of ambulance service geared towards betterment of health outcomes. It is recommended that EMS system should be designed ambulance services by considering the time period of the day and the future studies focus on the quality aspect and health outcomes of ambulance service use in pre-hospital conditions.

## Data Availability

The dataset used for the current studies are available and could be obtained from the correspondent author at any time upon reasonable request.

## Abbreviation

AOR: Adjusted Odds Ratio
CI: Confidence Interval
COR: Crude Odds Ratio
ED: Emergency Department
EMS: Emergency Medical Service
MOH: Ministry of Health
LMIC: Low- and Middle-Income Country
PHC: Pre-hospital Care
RTC: Road Traffic Crash
SSA: Sub-Saharan Africa
WHO: World Health Organization
